# Protein-Flavonoid Interaction Studies by a Taylor Dispersion Surface Plasmon Resonance (SPR) Technique: A Novel Method to Assess Biomolecular Interactions

**DOI:** 10.3390/bios6010006

**Published:** 2016-02-25

**Authors:** Preejith P. Vachali, Binxing Li, Brian M. Besch, Paul S. Bernstein

**Affiliations:** Moran Eye Center, University of Utah School of Medicine, 65 Mario Capecchi Drive, Salt Lake City, UT 84132, USA; preejith.vachali@hsc.utah.edu (P.P.V.); binxing.li@hsc.utah.edu (B.L.); brian.besch@hsc.utah.edu (B.M.B.)

**Keywords:** flavonoids, age-related macular degeneration, nutraceutical, taylor dispersion, human serum albumin, glutathione s-transferase pi isoform-1

## Abstract

Flavonoids are common polyphenolic compounds widely distributed in fruits and vegetables. These pigments have important pharmacological relevance because emerging research suggests possible anti-cancer and anti-inflammatory properties as well other beneficial health effects. These compounds are relatively hydrophobic molecules, suggesting the role of blood transport proteins in their delivery to tissues. In this study, we assess the binding interactions of four flavonoids (kaempferol, luteolin, quercetin, and resveratrol) with human serum albumin (HSA), the most abundant protein in the blood, and with glutathione S-transferase pi isoform-1 (GSTP1), an enzyme with well-characterized hydrophobic binding sites that plays an important role in detoxification of xenobiotics with reduced glutathione, using a novel Taylor dispersion surface plasmon resonance (SPR) technique. For the first time, HSA sites revealed a high-affinity binding site for flavonoid interactions. Out of the four flavonoids that we examined, quercetin and kaempferol showed the strongest equilibrium binding affinities (*K*_D_) of 63 ± 0.03 nM and 37 ± 0.07 nM, respectively. GSTP1 displayed lower affinities in the micromolar range towards all of the flavonoids tested. The interactions of flavonoids with HSA and GSTP1 were studied successfully using this novel SPR assay method. The new method is compatible with both kinetic and equilibrium analyses.

## 1. Introduction

Flavonoids are a group of plant-derived secondary metabolites. They are the most common group of polyphenolic compounds present in the human diet. These compounds are widely distributed in fruits, vegetables, nuts, tea, and wine [1]. Recently, dietary flavonoids have received increased attention, as they may potentially serve a protective role against a variety of diseases, such as cardiovascular diseases and certain cancers [2,3,4], and many researchers have been particularly interested in their potential protective roles against major eye diseases. One recent study has shown the protective effect of resveratrol by the inhibition of vascular endothelial growth factor (VEGF) secretion, a major angiogenesis signal protein that is a key mediator of the neovascular form of age-related macular degeneration (AMD) [5]. Flavonoids, mainly quercetin and its derivatives, are inhibitors of aldose reductase, an enzyme responsible for elevated polyols within the lens in diabetic cataracts [6]. Flavonoids are also known to protect retinal pigment epithelial cells and retinal ganglion cells from oxidative stress, a major contributor of many neurodegenerative diseases including AMD [7,8,9]. Kaempferol is known to attenuate the accumulation of the aging marker lipofuscin [10], and a protective effect of flavonoids against N-retinylidene-N-retinyl-ethanolamine (A2E) and light-induced photoreceptor death has been reported in primary bovine retinal cell culture [11].

Human serum albumin (HSA) is the most abundant extracellular protein present in the blood plasma at a concentration of 30–50 mg/mL [12]. It is a globular protein with three sub-units (I, II, and III), each containing two subdomains (A and B) [12]. It has been well documented that HSA possesses multiple binding sites, which allow it to interact with a variety of organic and inorganic molecules reversibly. It acts as a primary carrier of a variety of pharmaceutically relevant compounds in the human system. Thus, the study of the interactions between these small molecules and human serum albumin is important in understanding the distribution and metabolism of these small molecules to their target sites.

Glutathione S-transferase pi isoform-1 (GSTP1) belongs to the family of enzymes that plays a significant role in the detoxification of many electrophilic compounds with reduced glutathione. Thus, GST proteins have become important targets for development of inhibitors against metabolism of chemotherapeutic drugs. These enzymes are overexpressed in tumor cells, potentially resulting in multidrug resistance, and Zanden *et al* have reported an inhibitory effect of quercetin towards GSTP1 [13], so detailed characterization of binding interactions of these flavonoids with GST enzymes could provide information vital for the development of improved chemotherapeutic drugs. Moreover, our laboratory has identified GSTP1 as a zeaxanthin-binding protein in the macular region of human retina [14], and further studies of the potentially antagonistic or synergistic binding interactions of flavonoids and carotenoids with GSTP1 could provide insights on whether combination therapy with these two classes of bioactive nutrients would have beneficial or detrimental effects.

Surface plasmon resonance (SPR) technology has emerged as a powerful technique to study the interaction of small molecules with their respective target proteins because it provides reproducible, real-time information on ligand-binding interactions. Conventional SPR employs a series of standard fixed-concentration injections when analyzing biomolecular interactions to estimate kinetic parameters. This method is time-consuming and subject to various systematic errors [15]. Several new methods have been proposed to increase the throughputs and screening methods. For example, one-shot kinetics and kinetic titration methods have been reported [16,17], but even these methods have limitations including ligand denaturation due to long injection time and frequent regenerations, injection volume variations, and sample evaporation losses, which could result in poor data quality [15]. In this investigation, we have used an optimized Taylor dispersion SPR technique that can provide improved data output by reducing the systematic errors in standard injection methods.

## 2. Experimental Section

### 2.1. Materials

**Figure 1:**
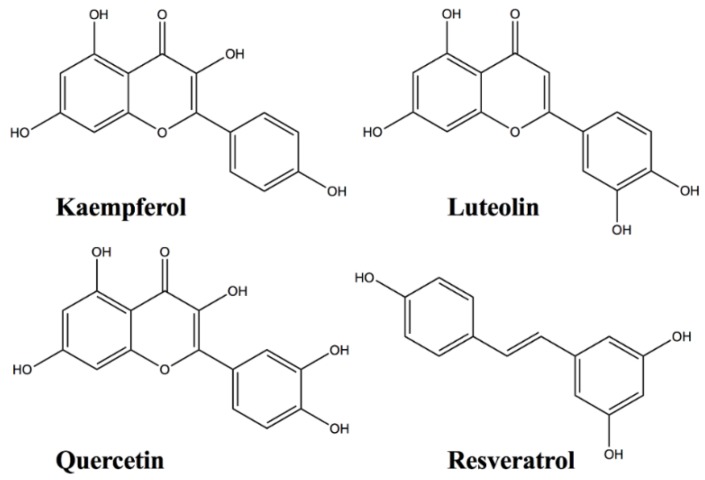
Chemical structure of the flavonoids used in this study.

**Figure 2:**
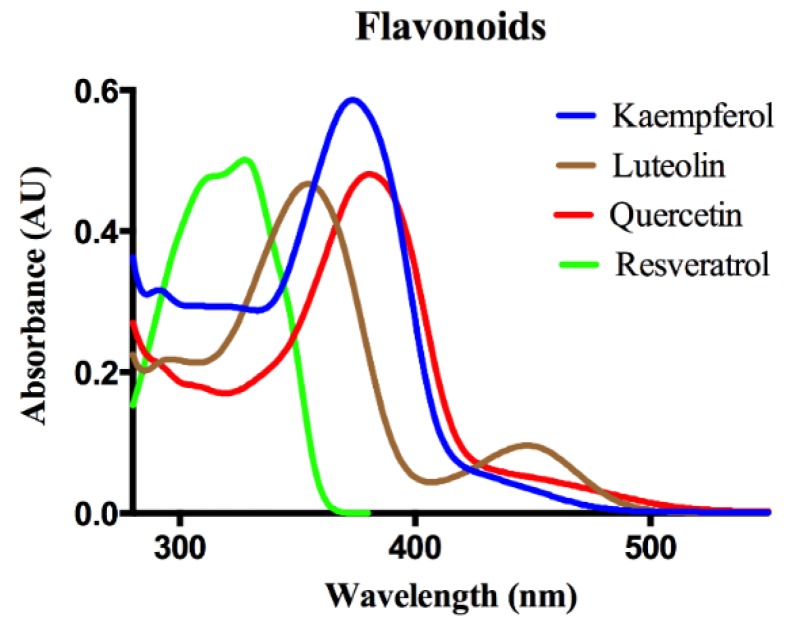
UV-Visible spectral signatures of tested flavonoids in methanol.

Hydroxyl-gel modified sensor chips and coupling reagents for the Pioneer SPR instrument (SensiQ Technologies, Oklahoma City, OK, USA) were obtained from Xantec (Duesseldorf, Germany). Figure 1 shows the chemical structures, and Figure 2 shows the UV-VIS spectra of the flavonoids (Sigma Aldrich, St. Louis, MO, USA) used in this study. All analyses were performed at 25 °C. 10 mM HEPES (pH 7.4) with 150 mM NaCl, 0.005% Tween-20, 1.26 mM EDTA, and 5% DMSO was used as the running buffer.

HSA (essentially fatty acid free; Sigma Aldrich, St. Louis, MO, USA) and GSTP1 (Fitzgerald Industries International, North Acton, MA, USA) (50 μg/mL in 10 mM sodium acetate, pH 5.0) were immobilized on polycarboxylate hydrogel sensor chip surfaces using a standard amine-coupling protocol (flow rate of 10 μL/min) to obtain a density of 10–12 kRU [18]. Each of the four flavonoids was dissolved in DMSO to achieve a high concentration, then further diluted to a final 5% DMSO concentration in running buffer. Typically, the flavonoid concentration series spanned 0.01–500 μM. Five blanks were analyzed at the beginning of the analysis, and the remaining blanks were randomly injected throughout the analysis for double-referencing purposes [19].

### 2.2. SPR Measurements

In this study, we explored the SensiQ Pioneer’s dynamic SPR capability (also known as the *OneStep^®^* injection method), which is essentially a Taylor dispersion to create a gradient injection to quantify biomolecular interactions [15]. In the Taylor dispersion method, analyte concentration is initially uniform, but it gradually changes into a sigmoidal gradient due to the combined actions of analyte diffusion and convective laminar flow within the dispersion capillary. This method saves sample preparation time, lessens the need for limited-supply sample materials, and eliminates possible human errors in the preparation of sample dilutions. Since the compounds are screened over a broader dilution range, content-rich data can be obtained from the preliminary screening step itself, and oftentimes, secondary screening can be avoided. A Taylor dispersion gradient injection of sucrose (3% w/v in running buffer) was performed in parallel as a diffusion standard to account for experimental deviations such as buffer viscosity, flow rate, temperature, *etc*. The analytes were injected in running buffer using the same Taylor diffusion gradient injection mode with a flow rate of 25 μL/min.

For assay validation, a conventional SPR method was used as a comparison [19,20,21,22]. The interaction of HSA with quercetin was chosen for validation. For the conventional SPR method, analyte dilutions were prepared manually in a twofold dilution series starting with 500 μM as the highest concentration. Each concentration series was injected in quadruplicate under similar buffer conditions that were used for the *OneStep^®^* assay.

### 2.3. Data Processing

SPR response data (sensorgrams) were zeroed at the beginning of each injection and double referenced [19]. For kinetic analyses, the responses were plotted against the analyte concentration and fit to a 1:1 (A + B = AB) or 1:2 (A + 2B = AB1 + AB2) binding model using Qdat analysis software (SensiQ Technologies, Inc., Oklahoma City, OK, USA) and GraphPad Prism 5.04 (GraphPad Software Inc., La Jolla, CA, USA).

## 3. Results and Discussion

**Figure 3:**
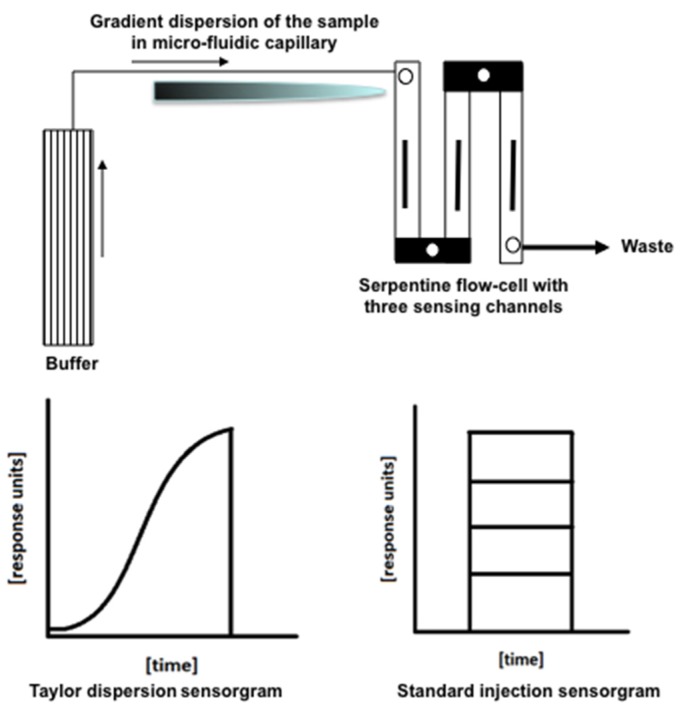
Schematic representation of the Taylor dispersion (*OneStep^®^*) assay method.

The newly available Taylor dispersion SPR assay method was successfully used in this study to reduce assay complexity and time. One of the major advantages of this method is that, unlike the standard fixed concentration method, analyte flows continuously over the surface in a slow gradient, which is equivalent to thousands of standard injection dilutions. Figure 3 shows the schematics of the Taylor dispersion injection pattern in comparison to the standard SPR injection method. The Taylor dispersion method was validated for HSA-warfarin interaction by Quinn, who reported no significant differences between it and the more commonly used standard injection method, and a simulation was also done to verify this concept [15,23]. Sample dilution is an automated process within the instrument, which further reduces systematic errors and simplifies processing methods.

### Protein-Flavonoid Interactions

We were able to immobilize 10–12,000 RU of HSA on the surface using amine coupling. Since there have been reports of instability of newly immobilized HSA surfaces, the surface was stabilized by injecting buffer overnight before starting the actual interaction studies [21].

**Figure 4:**
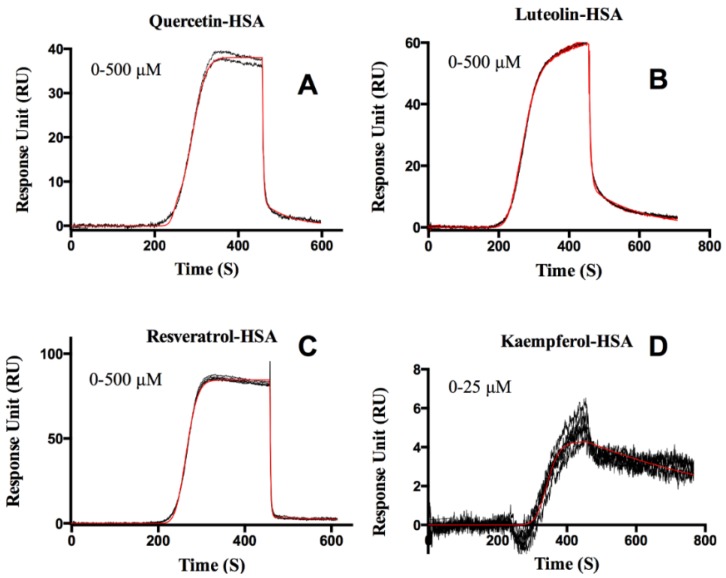
Sensorgrams of flavonoids (quercetin, luteolin, resveratrol, and kaempferol) interacting with HSA (Panels **A**–**D**). The orange lines show global kinetic analysis model fit to the response data to extract binding constants using a 1:2 model (Panels **A**-**C**) and a 1:1 model for panel **D**. The concentration range tested is indicated in each panel. The binding constants determined from the fits are listed in Table 1.

Kinetic constants are summarized in Table 1. Quercetin, luteolin, and resveratrol fit well to a 2-site model, while kaempferol fit to a 1-site model, as shown in Figure 4. Among the four flavonoids studied, kaemepferol showed the strongest affinity (37 ± 0.07 nM) towards HSA, followed by quercetin (63 ± 0.03 nM), resveratrol (400 ± 0.10 nM), and luteolin (63.40 ± 0.01 μM). Structural differences between the compounds probably account for variations in binding affinities of the four flavonoids tested.

**Figure 5:**
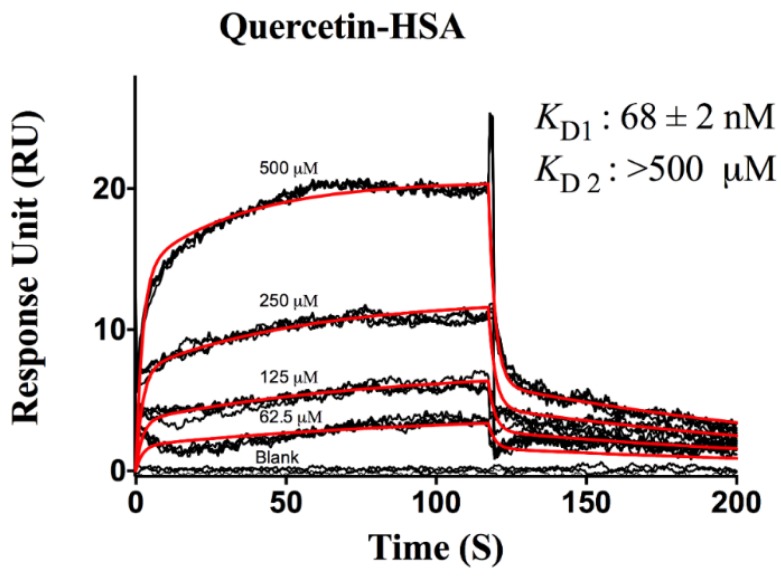
Sensorgram of quercetin interacting with HSA using the standard SPR injection method. The analytes were injected across the HSA surface in a two fold dilution series starting at 500 μM. The orange lines show a global fit to the response data used to extract binding constants using a 1:2 kinetic analysis model. The binding constants are reported in the insets.

The interaction of HSA with quercetin using the *OneStep^®^* assay was compared against the widely accepted standard injection method in order to validate the assay. Figure 5 shows a standard injection sensorgram obtained for HSA-quercetin interaction. Each dilution series was injected in quadruplicate, and the data were fitted using a 1: 2 interaction model to derive the binding constants. In the conventional SPR assay, quercetin displayed an affinity of 68 ± 2 nM towards HSA which compares favorably with the *OneStep^®^* *K*_D_ of 63 ± 0.03 nM. Both methods detected a low affinity site with *K*_D_ > 500 μM, the highest concentration tested.

Although there have been several studies done in the past on the interactions between HSA and flavonoids [24,25,26,27,28], their reported binding constants were much higher than ours which could be explained in retrospect because they were not able to resolve the high-affinity binding sites from other lower-affinity sites. In our SPR assay method, because of its high-resolution isotherm, we are able to characterize the high-affinity binding of flavonoids with HSA for the first time. Our results suggest that caution may be warranted when using flavonoid supplementations in those who are taking drugs like warfarin, as these important drugs could be outcompeted and displaced by high-affinity flavonoid molecules [29]. Also, in people who take flavonoid supplements regularly, it is important to know the optimal binding levels, as higher concentrations of these compounds could alter the structure of its binding protein and could even denature its activity. For example, Kanakis *et al.* observed that flavonoid complexation could cause protein unfolding at high concentration, due to the reduction of protein α-helical structure upon flavonoid interaction [30].

**Figure 6:**
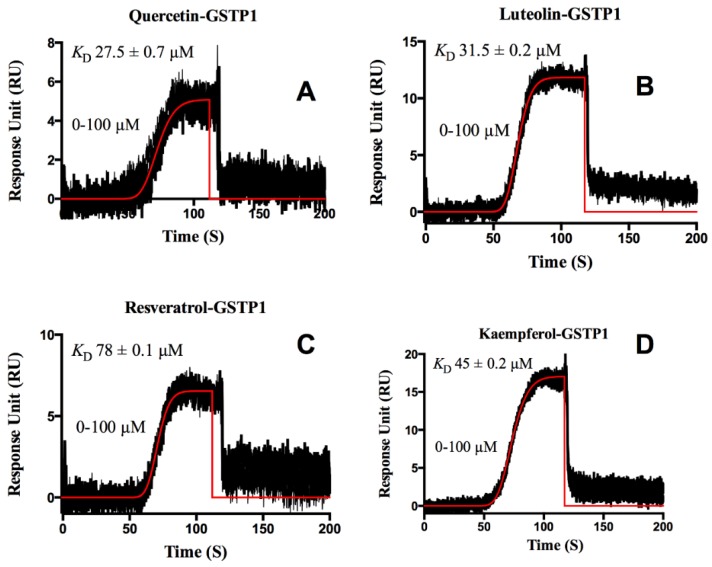
Sensorgrams of flavonoids interacting with GSTP1 (Panels **A**–**D**). The orange lines show a global fit to the response data used to extract binding constants using a 1:1 equilibrium analysis model. The concentration range tested is indicated in each panel. The *K*_D_ obtained from equilibrium analysis is also indicated in each panel.

In addition to HSA, we also studied GSTP1 whose interactions with flavonoids were relatively weak (micro-molar level), and there was only one binding site. Since the dissociation rate was fast, we did an equilibrium analysis to calculate the *K*_D_ (Figure 6). Zanden *et al.* reported that quercetin could potentially inhibit GSTP1, and it was found to be reversible [13]. In our study, we found that the GSTP1-quercetin interaction has a *K*_D_ of around 27.5 ± 0.7 μM, and we recorded similar binding affinities for the other flavonoids that we tested. Since flavonoids have a variety of beneficial effects against many diseases, these binding interactions studies will help in the development of clinically useful medicinal formulations of these compounds.

The Taylor dispersion assay method provides fast and accurate kinetic information on the binding interactions. This rapid analytical technique is particularly useful in high-throughput fragment screening for drug discovery. The binding affinities can be obtained from the primary screen itself, which eliminates the need for a secondary screening. Quinn reported that kinetic parameter determination using the Taylor dispersion method was not affected by non-specific binding up to 70% when carbonic anhydrase II-furosemide interactions were studied [23]. The effect of conformational changes on binding parameters can also be monitored in real-time using this method [23]. The apparent diffusion coefficient that can be calculated using the Taylor dispersion method can be used to understand analyte aggregation, denaturation, and other analyte artifacts [15]. This enhanced biophysical characterization technique is a useful alternative to the standard injection method in routine surface plasmon resonance analysis of biomolecular interactions.

## 4. Conclusions

The interactions of flavonoids with HSA and GSTP1 were studied successfully using the new SPR assay method. HSA interacted very strongly with the flavonoids that we tested. Since human serum albumin is the most abundant protein present in our blood serum, it is very important to understand its role in the transport and metabolism of small, hydrophobic molecules such as flavonoids. The Taylor dispersion gradient method afforded increased confidence in the high affinity site of HSA for flavonoids and in the assessment of the stoichiometry of flavonoid binding against both protein targets. We compared the conventional SPR assay with the *OneStep^®^* method, and similar binding affinities were obtained for both injection methods. The method is compatible with both kinetic and equilibrium analyses when certain kinetic criteria are met. Kinetic assays of this nature are critical in understanding the associated biological pathways of important dietary and pharmacological compounds.

## Figures and Tables

**Table 1:** Kinetic constants determined at 25 °C (HSA with flavonoids).

Flavonoids		*k*_a_ (M^−1^s^−1^)	*k*_d_ (s^−1^)	*K*_D_ (M)
Quercetin	Site 1	2.40 ± 0.01 × 10^5^	0.016 ± 0.003	6.30 ± 0.03 × 10^−8^
	Site 2	1.00 ± 0.10 × 10^2^	0.405 ± 0.004	>5.00 × 10^−4^
Luteolin	Site 1	3.20 ± 0.30 × 10^3^	0.205 ± 0.005	6.34 ± 0.01 × 10^−5^
	Site 2	1.71 ± 0.01 × 10^1^	0.007 ± 0.001	4.06 ± 0.02 × 10^−4^
Resveratrol	Site 1	7.00 ± 0.20 × 10^3^	0.003± 0.002	4.00 ± 0.10 × 10^−7^
	Site 2	2.90 ± 0.60 × 10^3^	0.520 ± 0.003	1.80 ± 0.40 × 10^−4^
Kaempferol	Site 1	4.84 ± 0.06 × 10^4^	0.002 ± 0.002	3.70 ± 0.07 × 10^−8^

*k*_a_: Association rate constant; *k*_d_: Dissociation rate constant; *K*_D_: Equilibrium dissociation constant. Numbers in parenthesis represent the standard error in the model fitting.
